# Editorial: Recent Advances in Endothelial Progenitor Cells Toward Their Use in Clinical Translation

**DOI:** 10.3389/fmed.2019.00290

**Published:** 2020-01-08

**Authors:** Reinhold J. Medina, David M. Smadja

**Affiliations:** ^1^School of Medicine, Dentistry, and Biomedical Sciences, Wellcome-Wolfson Institute for Experimental Medicine, Queen's University Belfast, Belfast, United Kingdom; ^2^Université de Paris, Innovative Therapies in Haemostasis, INSERM, Paris, France; ^3^Service d'Hématologie et Laboratoire de Recherches Biochirugicales (Fondation Carpentier), AH-HP, Georges Pompidou European Hospital, Paris, France

**Keywords:** endothelial progenitor, endothelial progenitor cell, endothelial colony forming cell, ECFC, vascular repair, therapeutic angiogenesis, angiogenesis

Cardiovascular disease remains the number one cause of death worldwide, by myocardial infarction, stroke, and critical limb ischemia. The common denominator in these diseases is the lack of blood vessels due to obstruction or degeneration. Therefore, the significant efforts for developing therapies based on endothelial progenitor cells (EPCs) for the regeneration of blood vessels in ischemic tissues. While preclinical evidence was convincing, results did not successfully translate into positive outcomes at clinical trials. Nevertheless, these first-in-man trials demonstrated safety and feasibility. The lack of efficacy is, to a certain extent, due to the poor cellular and molecular characterization of cells used, most of which were bone marrow mononuclear cells and described as enriched for endothelial progenitors. This fast clinical translation with ill-defined cell populations and negative results has damaged the field by dampening enthusiasm and hope from patients and clinicians.

We believe that cell therapies to regenerate blood vessels will be effective in future trials, but in order to achieve this, it is essential to clearly and unequivocally define the cells of interest, as required by next generation cytotherapies. Moreover, EPCs, more than a cell therapy product, have been proposed as a liquid biopsy to investigate endothelial dysfunction in patients with vascular diseases. This Research Topic presents 4 original research articles and 5 review articles aiming to update scientists surrounding our current understanding of endothelial progenitors, with emphasis on a well-defined subtype known as endothelial colony forming cells (ECFCs).

Keighron et al. provide a comprehensive up-to-date review of clinical trials using EPCs for diseases such as peripheral artery disease (PAD), coronary artery disease (CAD), ischemic stroke, and pulmonary artery hypertension (PAH). They concluded that EPCs used in clinical trials were highly heterogeneous cell populations and highlighted the need for better-defined cell populations such as ECFCs.

O'Neill et al. summarize preclinical evidence from various disease models to demonstrate the therapeutic potential of ECFCs for vascular repair in ischemic tissues.

The study by Tasev et al. explores in detail the response of ECFCs to hypoxia and their evidence indicates that 1% O_2_ impairs isolation, growth, and function in ECFCs.

Boisson-Vidal et al. report the role of Osteoprotegerin in enhancing ECFC differentiation from cord blood CD34+ cells.

Carolina et al. present results to underscore the detrimental effects of the glucocorticoid Dexamethasone on ECFCs by diminishing their wound healing ability through reduction of CXCR4.

The study by McLoughlin et al. identifies the optimal reference gene panel for PCR when investigating cellular senescence in ECFCs.

Rossi et al. summarize how Endoglin interfere with ECFCs vasculogenic properties and how it allows ECFCs involvement with inflammatory cells and hemostasis.

The review by Edwards et al. describe ECFCs and myeloid angiogenic cells (MACs) in inflammatory disorders including diabetes, rheumatoid arthritis, and systemic lupus erythematosus, with important insights into molecular mechanisms responsible for cellular dysfunction.

Pashalaki and Randi outline current uses of ECFCs from their application as tools to study disease such as von Willebrand disease, PAH, and diabetes, to their utilization to develop cell and gene therapies.

All the manuscripts presented in this Research Topic recognized the importance of harmonizing definitions and protocols, as well as advancing our knowledge in relation to the molecular understanding of ECFC biology, which is critical for successful translation into clinics. Better in-depth knowledge and detailed molecular characterization of ECFCs will enable our scientific community to finally build successful multi-centric clinical studies for biomarkers or therapeutic approaches, after more than 20 years of basic scientific discoveries.

## Author Contributions

RM and DS wrote and approved this editorial for publication.

### Conflict of Interest

The authors declare that the research was conducted in the absence of any commercial or financial relationships that could be construed as a potential conflict of interest.

